# *In vivo* imaging system (IVIS) therapeutic assessment of tyrosine kinase inhibitor-loaded gold nanocarriers for acute myeloid leukemia: a pilot study

**DOI:** 10.3389/fphar.2024.1382399

**Published:** 2024-05-10

**Authors:** Raluca-Andrada Munteanu, Adrian Bogdan Tigu, Richard Feder, Andra-Sorina Tatar, Diana Gulei, Ciprian Tomuleasa, Sanda Boca

**Affiliations:** ^1^ Medfuture Research Center for Advanced Medicine, Iuliu Hatieganu University of Medicine and Pharmacy, Cluj-Napoca, Romania; ^2^ Interdisciplinary Research Institute in Bio-Nano-Sciences, Babes-Bolyai University, Cluj-Napoca, Romania; ^3^ Molecular and Biomolecular Physics Department, National Institute for Research and Development of Isotopic and Molecular Technologies, Cluj-Napoca, Romania; ^4^ Department of Hematology, Ion Chiricuta Oncology Institute, Cluj-Napoca, Romania

**Keywords:** acute myeloid leukemia, tyrosine kinases, gold nanoparticles, drug delivery, *in vivo* models, *in vivo* imaging system

## Abstract

Acute myeloid leukemia (AML) is a malignancy in the myeloid lineage that is characterized by symptoms like fatigue, bleeding, infections, or anemia, and it can be fatal if untreated. In AML, mutations in tyrosine kinases (TKs) lead to enhanced tumor cell survival. The most frequent mutations in TKs are reported in *Fms*-like tyrosine kinase 3 (FLT3), Janus kinase 2 (JAK2), and KIT (tyrosine-protein kinase KIT), making these TKs potential targets for TK inhibitor (TKI) therapies in AML. With 30% of the mutations in TKs, mutated FLT3 is associated with poor overall survival and an increased chance of resistance to therapy. FLT3 inhibitors are used in FLT3-mutant AML, and the combination with hypomethylating agents displayed promising results. Midostaurin (MDS) is the first targeted therapy in FLT3-mutant AML, and its combination with chemotherapy showed good results. However, chemotherapies induce several side effects, and an alternative to chemotherapy might be the use of nanoparticles for better drug delivery, improved bioavailability, reduced drug resistance and induced toxicity. The herein study presents MDS-loaded gold nanoparticles and compares its efficacy with MDS alone, on both *in vitro* and *in vivo* models, using the FLT3-ITD-mutated AML cell line *MV-4-11 Luc2* transfected to express luciferin. Our preclinical study suggests that MDS-loaded nanoparticles have a better tumor inhibitory effect than free drugs on *in vivo* models by controlling tumor growth in the first half of the treatment, while in the second part of the therapy, the tumor size was comparable to the cohort that was treatment-free.

## 1 Introduction

Acute Myeloid Leukemia (AML) is a stem cell precursor malignancy in the myeloid lineage that can be fatal if untreated, with common symptoms such as fatigue, anemia, infections, or bleeding (Stubbins et al., 2022). In AML, the main tyrosine kinase (TK) mutations can lead to the upregulation of several biological pathways, which can enhance tumor cell survival (Wilson et al., 2018). TK mutations are mainly produced in *Fms-like tyrosine kinase 3* (FLT3), *Janus kinase 2* (JAK2), and KIT (*tyrosine-protein kinase KIT–CD117*); thus, some TK inhibitors (TKIs) could be used as targeted therapies (Altman et al., 2013). Mutations that occur in FLT3 account for 30% of all cases, mostly with FLT3 internal tandem duplication. These mutations in FLT3 are associated with the aggressiveness of AML, reduce overall survival (Kottaridis et al., 2001), and increase the chances of resistance to therapy and relapse (Rombouts et al., 2000). FLT3 inhibitors are promising therapeutic agents, with midostaurin (MDS) and gilteritinib approved by the FDA to be used in FLT3-mutant AML, both in first-line and salvage settings. Midostaurin was a path-breaker for gilteritinib; thus, the combination of FLT3 inhibitors with hypomethylating agents such as decitabine or azacitidine shows good results, and other FLT3 inhibitors are now undergoing clinical testing (Galanis et al., 2012; Levis, 2017; Perl, 2019; Smith, 2019). MDS is the first targeted therapy for AML with the FLT3 mutation, aiming to improve overall survival. MDS combined with chemotherapy showed good results in high-risk AML FLT3-mutant patients (Starr, 2016). RATIFY (NCT00651261), a phase III trial, showed that MDS addition to chemotherapy improves survival in FLT3-mutated patients (Stone et al., 2017). Although TKI first-line therapy is used for myelosuppression to induce a hematological response, several side effects like liver dysfunction, edema, fluid retention, fatigue, and gastrointestinal symptoms (Guilhot et al., 2012; Qosa et al., 2018; Mauro, 2021) might occur. On the other hand, chemotherapy usually induces serious side effects that augment in combination with other drugs by becoming acute. For example, the RADIUS_X study (NCT02624570) showed that half of the patients treated with midostaurin reported adverse events, with the most common being diarrhea and neutropenia (Roboz et al., 2020).

Chemotherapy alternatives tend to use nanoparticles for drug delivery to improve bioavailability and reduce drug resistance and toxicity (Yan et al., 2020). Our previous studies showed that FLT3 inhibitors loaded on Pluronic–gold nanoparticles inhibit tumor growth and have a superior therapeutic effect compared to bare drugs (Simon et al., 2015). In another study, we showed that FLT3 inhibitors loaded on gelatin-coated gold nanoparticles had an increased antitumor effect compared to the drug alone (Suarasan et al., 2016). Gold nanoparticles loaded with FLT3 inhibitors had an increased transmembrane delivery in AML cells, and the *in vitro* evaluation indicates that the *FLT3-IDT* gene was downregulated, leading to tumor cell suppression (Petrushev et al., 2016). Thus, MDS internalization in nanocarriers could improve its bioavailability and reduce its overall side effects.

In recent work, we have shown that the MDS loading and controlled release behavior greatly depend on the stimuli-responsive properties of the polymer that is conjugated onto a gold nanoparticle core (Tatar et al., 2023). Herein, we took a step forward by extending our studies toward preclinical *in vivo* investigations. For this, we employed a protocol based on data generated by an *in vivo* imaging system (IVIS) (Lim et al., 2009; Nakayama et al., 2020; Iluta et al., 2021), which allows us to track tumor cells and evaluate the drug effect in mice in a non-invasive manner over a longer time scale. Our findings show that the group treated with MDS-loaded nanoparticles had a better outcome than the group treated with MDS alone after 2 weeks of drug administration at a dosage approximately 10 times lower than the one given in clinical settings, considering the human-to-animal conversion. However, one can notice that the antitumor effect is more pronounced in the first half of the treatment, as after 4 weeks, the tumors have similar sizes in all treated groups. We attribute this effect to the significantly low concentration of MDS in loaded nanoparticles and, implicitly, to the overall dosage. Thus, further optimization of the nanoparticle formulation is a prerequisite for a better and more sustained outcome and the minimization of any potential relapse.

## 2 Materials

Hydrogen tetrachloroaurate (III) trihydrate (HAuCl_4_·3H_2_O, 99.99%), sodium citrate tribasic dihydrate, citric acid, Pluronic F-127 (Pluronic), midostaurin hydrate (>98%) (MDS), and L-glutathione reduced ≥98.0% (GSH) were obtained from Sigma-Aldrich. Thiolated polyethylene glycol of 5000 kDa molecular mass was obtained from Iris Biotech, Germany. The phosphate-buffered saline solution was purchased from Gibco^®^. All the other reagents used during the experiments are of analytical grade and were used without further purification. The aqueous solutions were prepared with ultrapure water (R = 18.2 MΩ cm) from a Milli-Q Purification System (Millipore, Merck). Ultrapure water was used for rinsing and cleaning procedures.

## 3 Methods

### 3.1 Gold nanoparticle synthesis and loading with the midostaurin drug

Gold nanoparticles were fabricated by chemical synthesis based on the Turkevich–Frens protocol (Boca et al., 2009). In brief, 100 mL of 1 mM HAuCl_4_·3H_2_O were heated until boiling. Under vigorous stirring, 10 mL of 38.8 mM sodium citrate were quickly added. During the boiling process, the solution changed color from yellow to an intense burgundy-red. Then, the solution was removed from the heat, and the stirring continued for another 10–15 min until the formation of nanoparticles. For the loading of the drug, 40 ul of midostaurin (1 mg/mL) were added to 1 mL of the synthesized nanoparticles in an aqueous solution, followed by the rapid addition of 2 mM Pluronic polymer to the nanoparticle–drug mixture. The mixture underwent 1 h of stirring at room temperature, after which the drug-loaded nanoparticles were purified by centrifugation and resuspension in phosphate-buffered saline (PBS). The amount of the drug that was loaded onto GNPs was obtained by measuring the drug content of the supernatant after particle purification. The loading efficiency (LE) was calculated using the following formula:

$$\%\text{LE}=\frac{T_{\text{Drug}}-S_{\text{Drug}}}{T_{\text{Drug}}}*100,$$

where T_drug_ is the total amount of the drug added and S_drug_ represents the amount of the drug in the supernatant (Simon et al., 2015). Drug loading was calculated by estimating the free drug content of the supernatant. The drug concentrations were determined by measuring the supernatant absorbance (area under the curve in the 300–380 nm range) and comparing it with a free drug solution calibration curve (ESI Supplementary Figure S1). Based on these, the drug content in the nanoparticle formulation was 30.534 μg/mL, while the gold quantity per particle was calculated to be 8.07E-17 g, considering the 3.67E+8 M extinction coefficient for 20-nm spherical nanoparticles. The purified drug loaded sample (GNP-MDS-PLU) was kept at 8°C until further use. The control sample of drug-free nanoparticles (GNP-PLU) was prepared by mixing the colloidal nanoparticles with Pluronic under similar conditions but by adding the PEG polymer as a particle pre-stabilizer before the Pluronic coating.

### 3.2 Drug release evaluation

To evaluate the release of the drug from the nanocarriers, the particles were re-suspended in buffer solutions mimicking the physiological environment (lysosomes and extracellular fluids): (i) acidic citrate buffer (pH 4.5); (ii) acidic citrate buffer (pH 4.5) containing a GSH; and (iii) PBS (pH 7.4). The nano-MDS samples were incubated at 37°C in the three buffers above for various time intervals: 1, 3, 8, and 24 h. At the selected time checkpoints, the samples were centrifuged to separate the nanoparticles from the supernatant containing the released drug molecules. The two fractions were spectroscopically measured for drug quantification, and the concentration of the released drug and that of the drug remaining within the nanoparticle were calculated based on calibration curves previously obtained for the MDS molecule. MDS quantification was based on the area under the curve measurements.

### 3.3 Physico-chemical characterization of the drug nanocarriers

UV-Vis-NIR extinction spectra of free and drug-loaded nanoparticles were acquired using a JASCO V-670 UV-Vis-NIR spectrometer at 1-nm spectral resolution in 2-mm path length quartz cuvettes. Particle size distribution and zeta potential were measured at 25°C using the Zetasizer Nano ZS90 from Malvern Instruments. Analysis was performed at a scattering angle of 90° and a temperature of 25°C. Particle morphology was imaged by transmission electron microscopy using a JEOL model JEM 1010 microscope.

### 3.4 Cell culture

The FLT3-ITD-mutated MV-4-11 AML cell line transfected to express luciferin MV-4-11 Luc2 (original cell line CRL-9591—MV-4-11) was cultured in sterile cell flasks at 37°C and 5% CO_2_ in a humidified chamber. The RPMI 1640 culture medium, supplemented with 10% fetal bovine serum (FBS), 1% penicillin/streptomycin, and 1% glutamine, was used for maintaining the MV-4-11 Luc2 cells under optimal growth conditions. All the cell culture reagents were purchased from Gibco.

### 3.5 Cell toxicity

The cell toxicity assay was performed using the CyQUANT XTT Cell Viability Assay Kit (Invitrogen, Carlsbad, CA, United States of America) with the experimental conditions set at 15 × 10^3 cells/well in a 100 µL culture medium in a sterile and flat bottomed 96-well plate. After 24 h of incubation, different concentrations of nanoparticles of various formulations (polymer and polymer–drug conjugates) were added to the cells. The cells were incubated with the treatment for another 24 h, followed by 4 h of incubation with the XTT reagent mixed with an electron-coupling reagent at 37°C, protected from light. The cell viability was measured at 450 nm and 660 nm using the Tecan Spark 10M spectrophotometer (TECAN, Austria GmbH, Grodig, Austria). Data analysis and graphical representation were performed using GraphPad Prism 8, and the result was expressed as the mean ± standard deviation (GraphPad Software, San Diego, CA, United States of America).

### 3.6 MV-4-11 Luc cell morphological analysis

The morphological changes in the cells and cell clustering after 24 h of treatment were evaluated in bright field using a Zeiss Axio Vert. A1 inverted microscope (Zeiss, Jena, Germany) with a ×5 objective. The images were captured using ZEN software (Zeiss, Jena, Germany).

### 3.7 *In vivo* protocols

Eight‐week‐old male and female athymic nude mice were included in the study. The mice were purchased from Charles River Laboratories and maintained in an authorized animal facility at the Medfuture Research Center for Advanced Medicine, Iuliu Hatieganu University of Medicine and Pharmacy, Cluj‐Napoca. The mice were accommodated at a standard temperature of 22°C ± 2°C and a relative humidity of 55% ± 10% in a 12:12 h light:dark cycle. The housing was made in an IVC2‐SM‐56‐IIL rack system (Acellabor) with individually ventilated cages supplied with HEPA‐filtered air (II L Cages) with autoclaved bedding and *ad libitum* access to autoclaved water and pelleted food. All experimental protocols were approved by the Ethics Committee of Iuliu Hatieganu University of Medicine and Pharmacy and were conducted in accordance with EU Directive 63/2010.

Sixteen mice were included in the study and tagged with metallic ear tags for identification. The animals were injected with approximately 2 × 10^6^ MV4-11 luciferase-positive cells in the knee joint while kept under gas anesthesia. The tumors were allowed to develop for 14 days, and at day 14, the size of the xenograft was measured using the IVIS SPECTRUM–IVIS Imaging System (PerkinElmer) using a systemically injected bioluminescent reporter optimized for *in vivo* imaging–RediJect D‐Luciferin (XenoLight, PerkinElmer). Out of the 16 mice, 11 developed relatively uniform tumors and were divided into four experimental groups as follows: control (2n), nano-control (3n), midostaurin control (3n), and nano-midostaurin (3n). We have chosen to include the fewest number of mice in the control group considering that the main purpose of the study was to demonstrate the superiority of the treatment loaded with nanoparticles compared with the standard free drug and not to demonstrate the feasibility of the actual treatment that was already tested in clinical trials. Control mice received 200 µL of buffer solution, nano-control mice received 200 µL of unloaded nanoparticles (GNP-PLU), midostaurin control mice received 200 µL of the equivalent dosage of a free drug, and nano-MDS mice received 200 µL of nanoparticles loaded with MDS in a PBS solution (GNP-MDS-PLU). All treatments were administered intraperitoneally (IP). The treatments were administered daily for 14 consecutive days, except for control mice that did not survive until the end of the experiment and received only seven doses of treatment. The evolution of the tumors was monitored *via* bioluminescent imaging at days 1, 8, and 15 for all treatment groups, except for the control mice that were followed until day 8. Bioluminescent images were processed using Living Image^®^4.5.2 software. The same software program was used to automatically measure the signal intensity within the region of interest (ROI) using the automatic contour tool.

## 4 Results and discussion

### 4.1 Characterization of midostaurin nanocarriers

**FIGURE 1:**
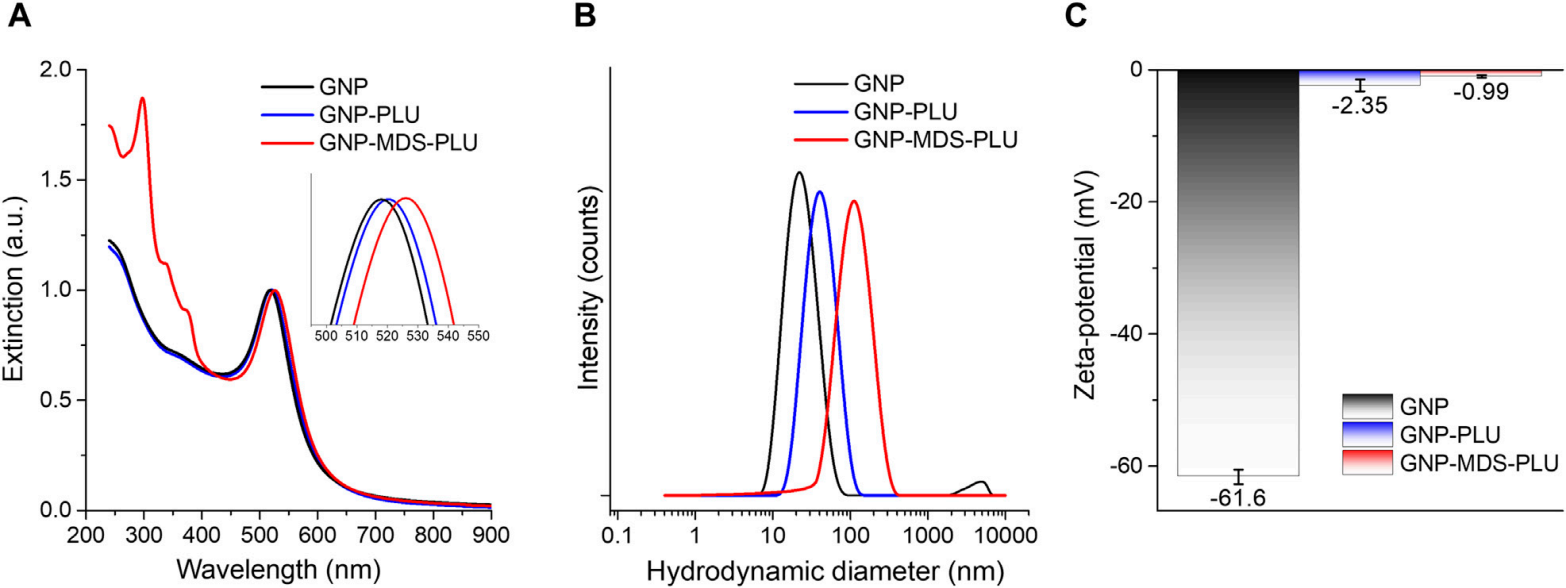
**(A)** UV-Vis-NIR extinction spectra of Pluronic nanoconjugates of various composition. Inset: LSPR (the spectra are normalized to unity). **(B)** DLS hydrodynamic diameter data of nanoconjugates. **(C)** Zeta potential values of nanoconjugates. Black, pristine GNPs; blue, Pluronic-conjugated nanoparticles; red, MDS-loaded nanoparticles.

The synthesized gold nanoparticles have a spherical shape and sizes of approximately 20 nm, as indicated by the morphological characterization of the TEM micrograph (ESI Supplementary Figure S2). The particles were purified by centrifugation and resuspension in ultrapure water and used for further functionalization. Figure 1 presents the UV-Vis-NIR spectra of the particles in different formulations (free, conjugated with Pluronic, and loaded with MDS drug). The characteristic peaks of midostaurin are visible in the 300–380 nm region. The nanoconjugates have a very high MDS loading efficiency (94.65%) facilitated by the midostaurin, which, being an organic hetero-octacyclic compound, is capable of participating in less than five hydrogen bonds, so it is mostly hydrophobic. Pluronic molecules are hydrophilic–hydrophobic–hydrophilic block copolymers of poly(ethylene oxide) (PEO) and poly(propylene oxide) (PPO). Taking these into consideration, the mechanism by which MDS-loaded gold nanoparticles are formed is based on the self-assembly of Pluronic and MDS into amphiphilic structures that engulf the nanoparticles suspended in an aqueous solution. The hydrophobic area of MDS is captured in the PPO layer of the Pluronic via hydrophobic interactions, and the outer hydrophilic shell ensures the solubility and stability of the system. The LSPR position is red-shifted by several nm after conjugation with the polymer and drug molecules, respectively, confirming that the drug and polymer molecules are conjugated onto the nanoparticle surface. DLS measurements corroborate the results, as the measured hydrodynamic diameter systematically increases after each round of functionalization from approximately 25 nm in the case of bare nanoparticles to 45 nm and 123 nm, respectively, for polymer and MDS-polymer-coated ones. The zeta potential also modifies, showing a value of −61.6 ± 1.1 mV for the pristine GNPs, while after Pluronic shielding, the value increases to −2.35 ± 0.9 mV. The presence of MDS in the nanoparticle formulation further modifies the zeta potential of the particles to values approaching neutral charge. Still, the nanoparticles remain stable in a biologically compatible buffer solution due to the presence of the polymer, which provides steric stability to the nanocomplexes.

### 4.2 Evaluation of the drug release profile

**FIGURE 2:**
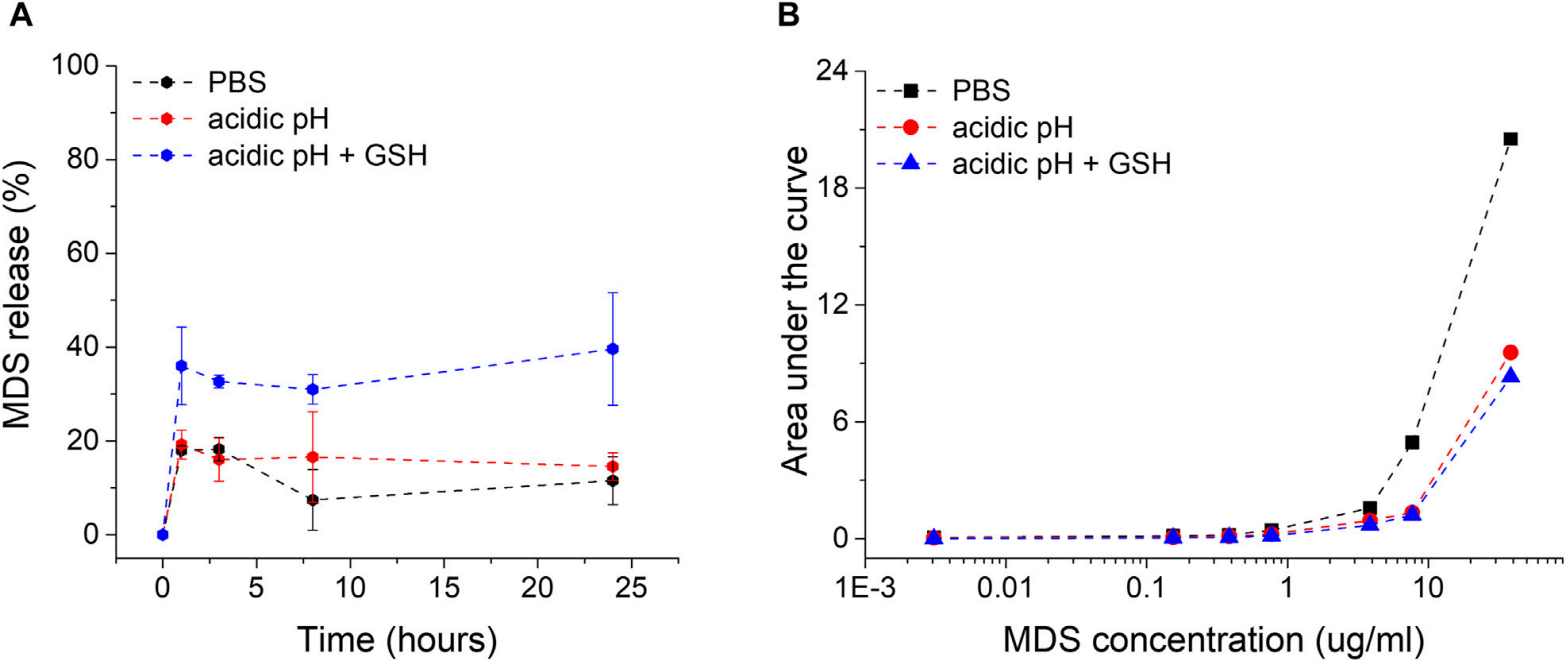
**(A)** Representation of the MDS drug release rates from midostaurin-loaded Pluronic conjugated gold nanoparticles (GNP-MDS-PLU) in function of time in various incubation media. **(B)** Calibration curves for MDS quantification in all three release media solutions (black, PBS; red, acidic pH; blue, GSH presence in acidic pH).

The particles were subjected to *ex vivo* release buffers simulating relevant intracellular microenvironment conditions. PBS is commonly used due to its resemblance to extracellular fluid pH of 7.4 and ionic strength. A citrate-based acidic buffer with a pH of 4.5 resembles lysosomal pH conditions, as GNPs are engulfed by cells mostly based on endocytosis through the endo-lysosomal internalization system. For an even more accurate simulation of the intra-lysosomal microenvironment where GNP-MDS-PLUs are located after internalization, we added 10 mM of glutathione (GSH), an important component of the lysosomal fluid and the most abundant thiol in animal cells. Due to its chemical composition, Pluronic is a pH-non-sensitive polymer, and Pluronic-coated nanoparticles are relatively stable under both PBS (7.4 pH) and acidic (pH 4.5) conditions (Figure 2). Similar results regarding the stability of conjugated nanoparticles in the PBS buffer were previously obtained and demonstrated in our group (Simon et al., 2015; Tatar et al., 2023). Herein, we expand the evaluation by testing the stability of the particles over a 3-day time period. The results indicate the high stability of the drug nanocomplex since both the particle LSPR band position and the spectral characteristics of the drug molecules are conserved (ESI Supplementary Figure S3). The drug release experiments showed a burst release in the first hour of incubation for all release buffers but barely reached 16%–17% MDS release after 24 h of incubation under non-GSH conditions. In contrast, the presence of glutathione leads to release rates of 50% after 24 h due to the molecules’ high affinity to the gold surface of the particles, leading to a major displacement of the adsorbed MDS and Pluronic (Figure 2). The released MDS is most likely captured in Pluronic micelles within the hydrophobic core.

### 4.3 *In vitro* cytotoxicity evaluation

**FIGURE 3:**
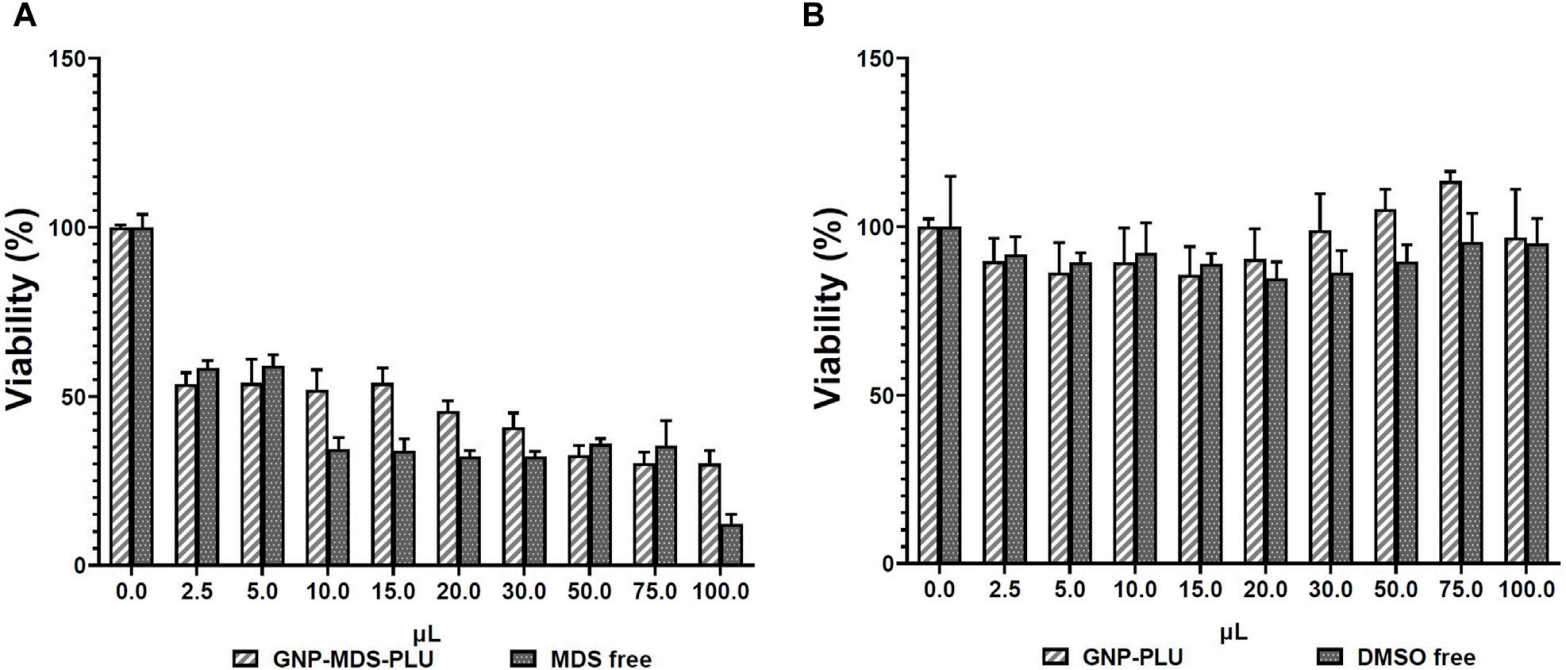
**(A)** Cytotoxicity evaluation by the XTT assay of GNP-MDS-PLU and MDS and **(B)** GNP-PLU and DMSO on MV-4-11 Luc2 cells after 24 h of treatment.

*In vitro* toxicity of MDS, GNP-PLU, and GNP-MDS-PLU on MV-4-11 Luc2 leukemia cells was evaluated at 24 h after treatment. The cytotoxicity was assessed at different concentrations of the above-mentioned nanocompounds by adding different volumes of the colloid containing either nanoparticles or the free drug in culture media (volume range 2.5–100 μL), as presented in Figures 3A, B. The GNP-PLU concentration as particles/mL was adapted to match that of GNP-MDS-PLU, which was loaded with MDS as an anti-FLT3 compound.

The MTS results indicate that the GNPs without drug load showed no toxicity on MV-4-11 Luc2 cells, while the MDS alone and GNP-MDS-PLU treatments inhibited leukemia cell growth in a dose-dependent manner. Furthermore, it is notable that at a very low dose of the free midostaurin drug and midostaurin-loaded nanoparticles, the inhibition effect is significant, downstreaming the viability by almost 50%. This aspect highlights the high antitumor effect of midostaurin. The reduced cell population was evaluated by bright-field microscopy, and the results are presented in the Supplementary Material attached to the article (ESI Supplementary Figure S4). The nonlinear regression fit of the obtained data indicates a low IC_50_ value, confirming that the compound can inhibit half of the MV-4-11 Luc2 cells at a very low dose in a short period of treatment of 24 h, while the unloaded GNPs showed no toxicity (ESI Supplementary Figure S5).

### 4.4 *In vivo* cytotoxicity evaluation

**FIGURE 4:**
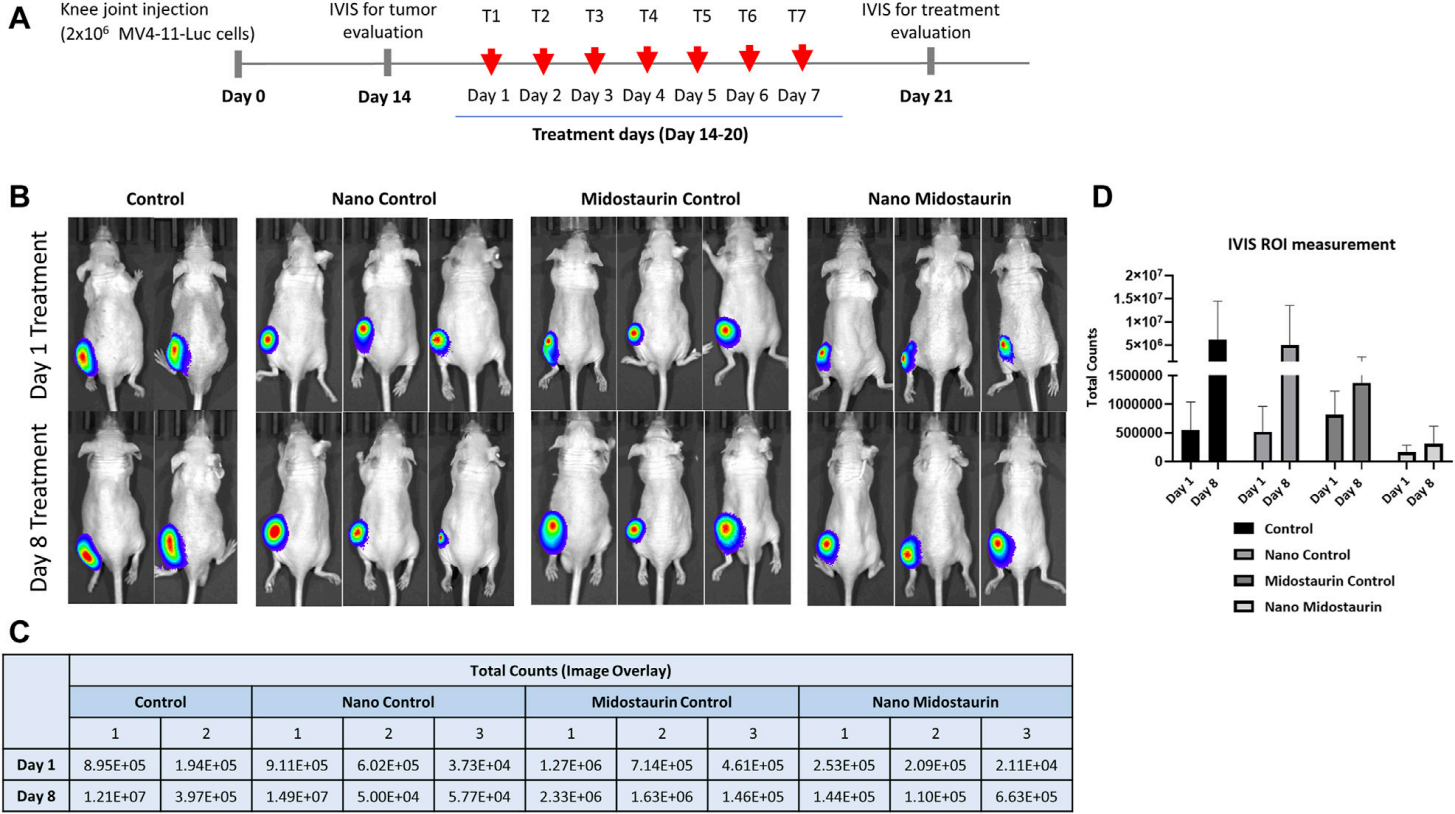
*In vivo* evaluation of systemic efficiency of various gold nanocarrier formulations—14 days of continued acute myeloid leukemia treatment. **(A)** Scheme of tumor development, treatment administration, and imagistic evaluation schedule. **(B)** *In vivo* bioluminescent evaluation of mice grafted with MV4-11-Luc cells in the knee joint divided into four experimental groups: control, nano-control, midostaurin control, and nano-midostaurin. *In vivo* imaging was done before treatment administration (day 1 of treatment) and after the last dose of treatment (day 8 of treatment). **(C)** Measurement of bioluminescent signal intensity within the region of interest of the tumor graft before treatment administration (day 1) and after the last dose of treatment (day 8) of the four experimental groups: control, nano-control, midostaurin control, and nano-midostaurin. **(D)** Graphical representation of the bioluminescent signal intensity of ROI according to the values presented at Figure 4C.

In order to demonstrate the improved efficiency of the treatment loaded into the particles in a preclinical experimental setup closer to the patients’ scenario, we used athymic nude mice with tumor xenografts as models for the evolution of the disease. Once we observed a significant tumor formation, we administered systemically via IP injection control buffer (control), free nanoparticles (nano-control), free drug (midostaurin control), and particles loaded with the drug (nano-midostaurin) (Figure 4). The treatment was administered daily for 14 days (ESI Supplementary Figure S6) and evaluated on days 1, 8, and 15 using the IVIS. The therapy delivery approach was chosen according to its pharmacokinetic and pharmacodynamic properties to ensure appropriate therapeutic plasma levels. The daily intraperitoneal dose for 14 consecutive days was chosen to ensure consistent exposure to the therapy, which is important to determine its therapeutic effect. This strategy was informed by existing literature data, suggesting that such a regimen could minimize toxicity while maximizing tumor inhibition (Lu et al., 2022). Moreover, we hypothesized that nanoparticles might exhibit delayed effects on tumor progression or interact differently with the tumor microenvironment over an extended period. The control group that did not receive any form of treatment died under anesthesia at the second IVIS evaluation (day 8), stopping the experiment on day 7 of treatment (Figure 4). As can be seen in Figure 4, in the case of control mice that received only buffer solution or nanoparticles alone, the xenografts developed significantly without any signs of tumor inhibition. In the third control group, meaning the mice that were injected with the free drug, the malignant formation decreased at day 8 compared with the other two control cohorts; however, the treatment did not manage to completely stop tumor evolution. Finally, when midostaurin was loaded into the particles and administered systemically (nano-midostaurin), we observed a significant improvement in terms of tumor development, where the new formulation managed to significantly minimize tumor growth compared with the other three control situations, including the free drug cohort.

Despite losing the control group in the first half of the experiment, we continued the protocol until day 14 of the treatment with the other three cohorts, considering that the main purpose of the preclinical evaluation consisted of demonstrating the improved efficiency of the new particles loaded with the drug compared with the drug alone. Moving past day 7 of treatment, we observed a sudden rapid tumor growth also in the nano-midostaurin cohort, similar to the one from the midostaurin control and nano-control groups (ESI Supplementary Figure S6). We attributed this change to the highly aggressive character of the tumor but also to the fact that, at this point, we were unable to load into the particles a clinically equivalent dose of midostaurin. To establish our dosing strategy, we used an approved therapeutic regimen, and we consulted the RATIFY clinical trial (Stone et al., 2017), where midostaurin was administered at 50 mg orally twice daily in adult AML patients with FLT3 mutations. Considering the pharmacokinetic and pharmacodynamic differences between humans and mice, we used a scaled dose that was approximately 10 times lower than the human equivalent dose when adjusted for the body surface area. This adjustment is consistent with standard practices for translating human doses to mouse doses, ensuring relevance and safety in our preclinical investigation. Hence, the dosage was calculated according to the human–animal dose conversion, being limited by the volume that can be administered to a mouse. However, the new therapeutic formulation has managed to keep the tumor under control for half of the therapeutic scheme, a fact that did not happen in the case of the standard treatment that is currently used in the clinic.

## 5 Discussions and future perspectives

Our preclinical study shows that midostaurin in the nanoparticle formulation has improved effects on tumor inhibition compared to the free drug *in vivo*, managing to keep tumor formation under control with no significant tumor growth within the first half of the treatment (ESI Supplementary Figure S7). However, starting with the second half of the therapy, the tumor growth intensified even under the new nanoparticle-based formulation, reaching similar levels to those observed in the free midostaurin cohort. We mainly attribute this spurt to an insufficient drug concentration, being limited by the volume of treatment that can be injected into a mouse and, implicitly, the particle concentration. It is, therefore, notable that the administered drug quantity with positive results was approximately 10 times lower than the one given in the clinic after the human-to-animal conversion. However, in the case of a patient, this limitation does not exist anymore since a much higher tolerance can be applied in terms of treatment volume. To overcome these limitations, we plan to conduct further research with an expanded sample size and investigate different statistical approaches. Furthermore, considering the challenges in maintaining consistent drug concentrations due to the limitations in treatment volume that can be administered in a mouse model, future studies will also focus on optimizing the nanoparticle formulation. This will improve the bioavailability and sustained release of midostaurin, potentially increasing the efficacy of the treatment throughout the entire research period.

## Data Availability

The raw data supporting the conclusion of this article will be made available by the authors, without undue reservation.

## References

[B1] Altman J. K. Szilard A. K. Platanias L. C. (2013). Tyrosine kinase inhibition in acute myeloid leukemia. Leukemia Lymphoma 54, 1351–1352. 10.3109/10428194.2012.754889 23206226

[B2] Boca S. C. Farcau C. Astilean S. (2009). The study of Raman enhancement efficiency as function of nanoparticle size and shape. Nucl. Instrum. Methods Phys. Res. Sect. B Beam Interact. Mater. Atoms 267, 406–410. 10.1016/j.nimb.2008.10.020

[B3] Galanis A. Rajkhowa T. Muralidhara C. Ramachandran A. Levis M. J. (2012). Crenolanib is A highly potent, selective, FLT3 TKI with activity against D835 mutations. Blood 120, 1341. 10.1182/blood.V120.21.1341.1341

[B4] Guilhot F. Hughes T. P. Cortes J. Druker B. J. Baccarani M. Gathmann I. (2012). Plasma exposure of imatinib and its correlation with clinical response in the tyrosine kinase inhibitor optimization and selectivity trial. Haematologica 97, 731–738. 10.3324/haematol.2011.045666 22315495 PMC3342976

[B5] Iluta S. Pasca S. Gafencu G. Jurj A. Terec A. Teodorescu P. (2021). Azacytidine plus olaparib for relapsed acute myeloid leukaemia, ineligible for intensive chemotherapy, diagnosed with a synchronous malignancy. J. Cell Mol. Med. 25, 6094–6102. 10.1111/jcmm.16513 34132464 PMC8406486

[B6] Kottaridis P. D. Gale R. E. Frew M. E. Harrison G. Langabeer S. E. Belton A. A. (2001). The presence of a FLT3 internal tandem duplication in patients with acute myeloid leukemia (AML) adds important prognostic information to cytogenetic risk group and response to the first cycle of chemotherapy: analysis of 854 patients from the United Kingdom Medical Research Council AML 10 and 12 trials. Blood 98, 1752–1759. 10.1182/blood.V98.6.1752 11535508

[B7] Levis M. (2017). Midostaurin approved for FLT3-mutated AML. Blood 129, 3403–3406. 10.1182/blood-2017-05-782292 28546144

[B8] Lim E. Modi K. D. Kim J. (2009). *In vivo* bioluminescent imaging of mammary tumors using IVIS spectrum. J. Vis. Exp. JoVE, 1210. 10.3791/1210 19404236 PMC2794084

[B9] Lu H. Weng X.-qin Sheng Y. Wu J. Xi H.-min Cai X. (2022). C*ombination of midostaurin and ATRA exerts dose-dependent dual effects on acute myeloid leukemia cells with wild type FLT3* . BMC Cancer 22, 749. 749. 10.1186/s12885-022-09828-2 35810308 PMC9270826

[B10] Mauro M. J. (2021). Lifelong TKI therapy: how to manage cardiovascular and other risks. Hematology 2021, 113–121. 10.1182/hematology.2021000239 34889360 PMC8791114

[B11] Nakayama J. Saito R. Hayashi Y. Kitada N. Tamaki S. Han Y. (2020). High sensitivity *in vivo* imaging of cancer metastasis using a near-infrared luciferin analogue seMpai. Int. J. Mol. Sci. 21, 7896. 10.3390/ijms21217896 33114327 PMC7660630

[B12] Perl A. E. (2019). Availability of FLT3 inhibitors: how do we use them? Blood 134, 741–745. 10.1182/blood.2019876821 31243041

[B13] Petrushev B. Boca S. Simon T. Berce C. Frinc I. Dima D. (2016). Gold nanoparticles enhance the effect of tyrosine kinase inhibitors in acute myeloid leukemia therapy. Int. J. Nanomedicine 11, 641–660. 10.2147/IJN.S94064 26929621 PMC4760658

[B14] Qosa H. Avaritt B. R. Hartman N. R. Volpe D. A. (2018). *In vitro* UGT1A1 inhibition by tyrosine kinase inhibitors and association with drug-induced hyperbilirubinemia. Cancer Chemother. Pharmacol. 82, 795–802. 10.1007/s00280-018-3665-x 30105461

[B15] Roboz G. J. Strickland S. A. Litzow M. R. Dalovisio A. Perl A. E. Bonifacio G. (2020). Updated safety of midostaurin plus chemotherapy in newly diagnosed FLT3 mutation-positive acute myeloid leukemia: the RADIUS-X expanded access program. Leuk. Lymphoma 61, 3146–3153. 10.1080/10428194.2020.1805109 32812818

[B16] Rombouts W. J. C. Blokland I. Löwenberg B. Ploemacher R. E. (2000). Biological characteristics and prognosis of adult acute myeloid leukemia with internal tandem duplications in the Flt3 gene. Leukemia 14, 675–683. 10.1038/sj.leu.2401731 10764154

[B17] Simon T. Tomuleasa C. Bojan A. Berindan-Neagoe I. Boca S. Astilean S. (2015). Design of FLT3 inhibitor - gold nanoparticle conjugates as potential therapeutic agents for the treatment of acute myeloid leukemia. Nanoscale Res. Lett. 10, 466. 10.1186/s11671-015-1154-2 26625890 PMC4666845

[B18] Smith C. C. (2019). The growing landscape of FLT3 inhibition in AML. Hematol. Am. Soc. Hematol. Educ. Program 2019, 539–547. 10.1182/hematology.2019000058 PMC6913436 31808872

[B19] Starr P. (2016). Midostaurin the first targeted therapy to improve survival in AML: potentially practice-changing. Am. Health Drug Benefits 9, 1–21. PMC4782225 27014400

[B20] Stone R. M. Mandrekar S. J. Sanford B. L. Laumann K. Geyer S. Bloomfield C. D. (2017). Midostaurin plus chemotherapy for acute myeloid leukemia with a FLT3 mutation. N. Engl. J. Med. 377, 454–464. 10.1056/NEJMoa1614359 28644114 PMC5754190

[B21] Stubbins R. J. Francis A. Kuchenbauer F. Sanford D. (2022). Management of acute myeloid leukemia: a review for general practitioners in oncology. Curr. Oncol. 29, 6245–6259. 10.3390/curroncol29090491 36135060 PMC9498246

[B22] Suarasan S. Simon T. Boca S. Tomuleasa C. Astilean S. (2016). Gelatin-coated gold nanoparticles as carriers of FLT3 inhibitors for acute myeloid leukemia treatment. Chem. Biol. Drug Des. 87, 927–935. 10.1111/cbdd.12725 26808072

[B23] Tatar A.-S. Nagy-Simon T. Tigu A. B. Tomuleasa C. Boca S. (2023). Optimization of tyrosine kinase inhibitor-loaded gold nanoparticles for stimuli-triggered antileukemic drug release. J. Funct. Biomater. 14, 399. 10.3390/jfb14080399 37623644 PMC10455807

[B24] Wilson L. J. Linley A. Hammond D. E. Hood F. E. Coulson J. M. MacEwan D. J. (2018). New perspectives, opportunities, and challenges in exploring the human protein kinome. Cancer Res. 78, 15–29. 10.1158/0008-5472.CAN-17-2291 29254998

[B25] Yan L. Shen J. Wang J. Yang X. Dong S. Lu S. (2020). Nanoparticle-based drug delivery system: a patient-friendly chemotherapy for oncology. Dose Response 18, 1559325820936161. 10.1177/1559325820936161 32699536 PMC7357073

