# Supplementary material for: In vivo imaging system (IVIS) therapeutic assessment of tyrosine kinase inhibitor-loaded gold nanocarriers for acute myeloid leukemia: a pilot study
Source: Front Pharmacol. 2024 May 10;15:1382399. doi: 10.3389/fphar.2024.1382399 (PMC11116693; doi:10.3389/fphar.2024.1382399)
Supplement: Supplementary file 1 [file DataSheet1.pdf]

In vivo imaging system (IVIS) therapeutic assessment of tyrosine kinase inhibitor loaded gold nanocarriers for acute myeloid leukemia: A pilot study

Raluca-Andrada Munteanu[1†], Adrian Bogdan Tigu[1†], Richard Feder[1], Andra-Sorina Tatar[2,3], Diana Gulei[1,*], Ciprian Tomuleasa[1,4,*] Sanda Boca [2,3,*]

[1] *Medfuture Research Center for Advanced Medicine, Iuliu Hatieganu University of Medicine and Pharmacy, 400349 Cluj Napoca, Romania*

[2] *Interdisciplinary Research Institute in Bio-Nano-Sciences, Babes-Bolyai University, 400271 Cluj-Napoca, Romania*

[3] *Molecular and Biomolecular Technologies Department, National Institute for Research and Development of Isotopic and Molecular Technologies, 400293 Cluj-Napoca, Romania*

[4] *Department of Hematology, Ion Chiricuta Oncology Institute, 400124 Cluj Napoca, Romania*

*corresponding authors
†these authors contributed equally to this work

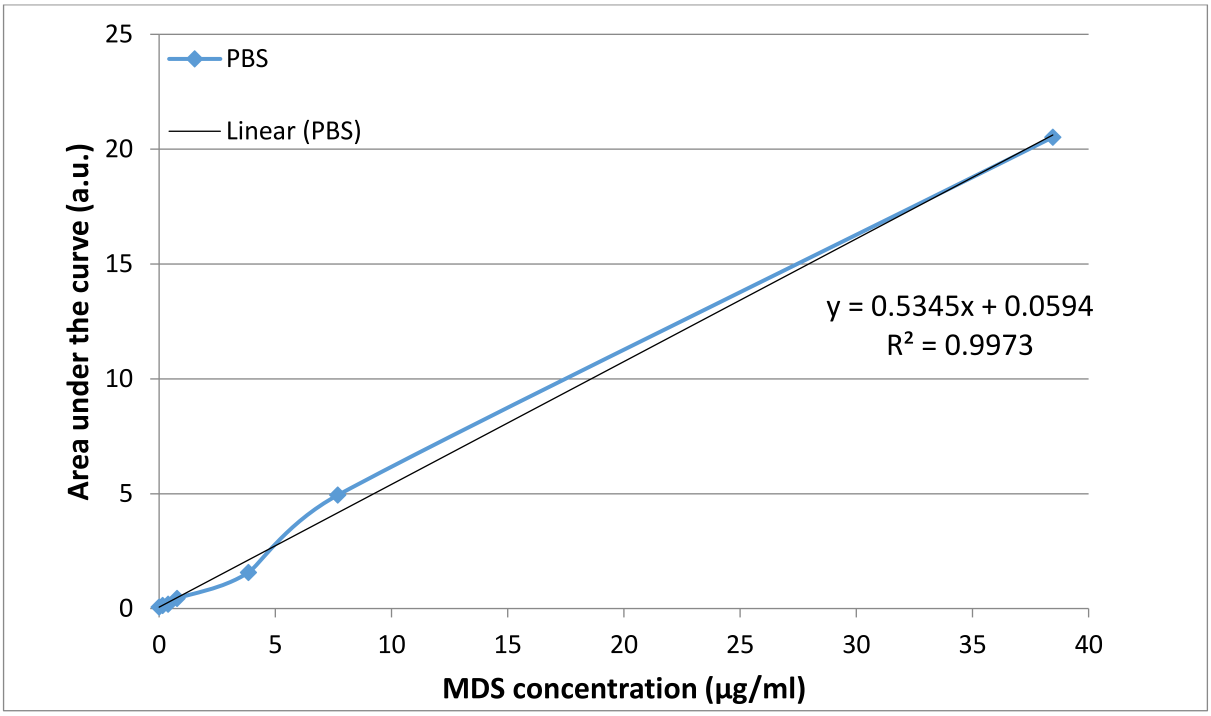

Figure S1. Calibration curve of Midostaurin (MDS), expressed as MDS area under the curve in the 300-380 nm spectral region, as a function of MDS concentration (µg/ml). Inset: Linearity equation and the R-squared.

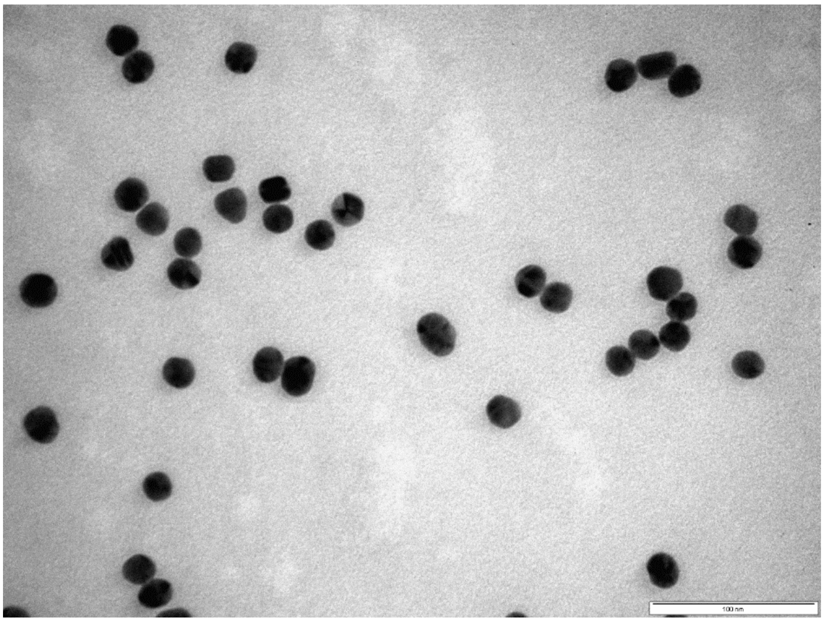

Figure S2: Transmission electron microscopy image of 20 nm gold nanoparticles used as template for the design of Midostaurin loaded nanocarriers. Scale bar: 100 nm.

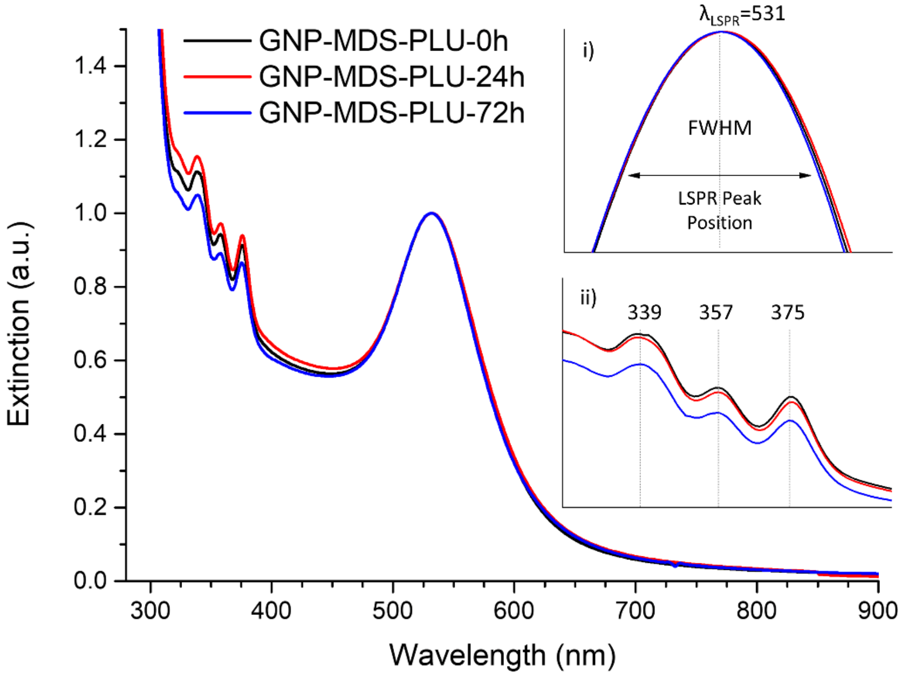

Figure S3. UV-Vis-NIR extinction spectra of the GNP-MDS-PLU nanoparticles in PBS medium, recorded over 3 days. Inset: i) detail on the normalized LSPR band; ii) detail on the MDS drug characteristic bands; FWHM - full width at half maximum.

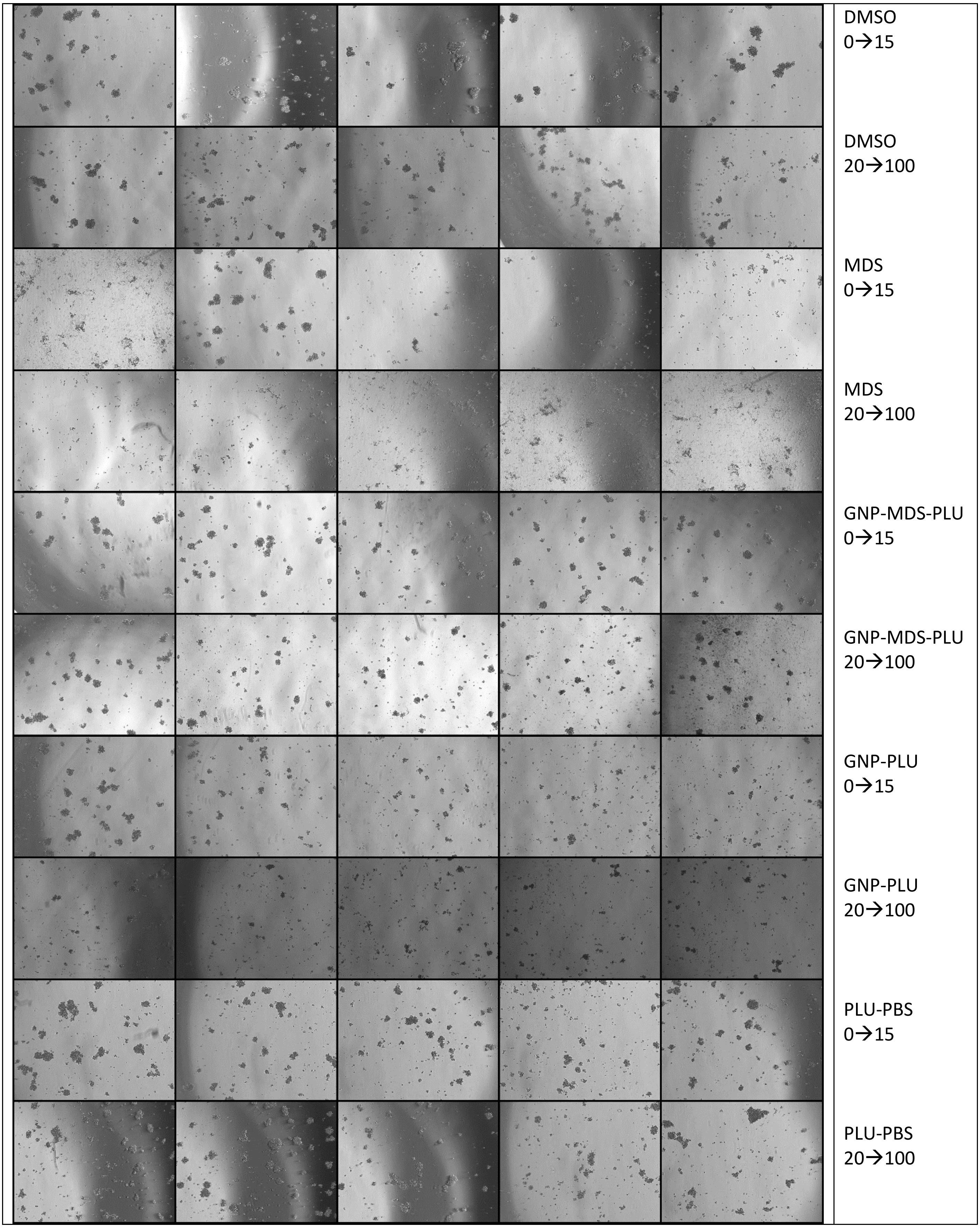

Figure S4: Bright field microscopy images of MV-4-11 Luc2 cells incubated with different volumes (0-100 ul) of the treatment compounds.

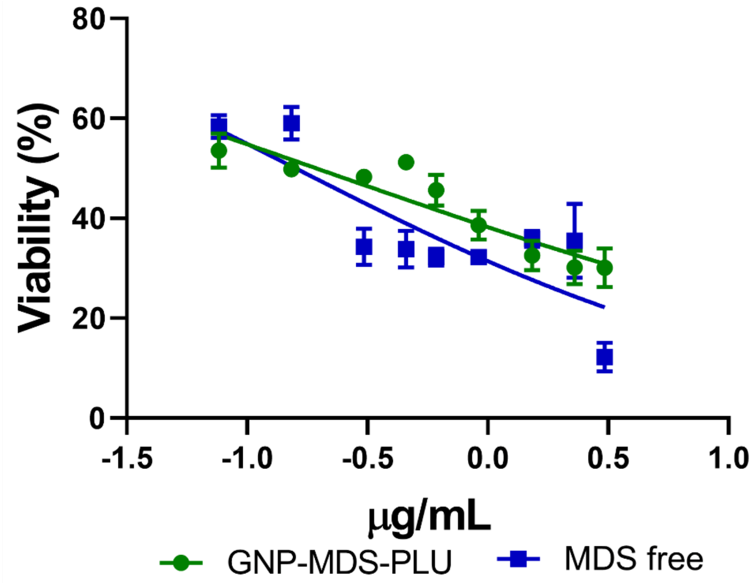

Treatment quantity-volume **(µL)**	Treatment quantity-equivalent mass **(µg)**
0	**0**
2.5	**0.0763**
5	**0.1526**
10	**0.3053**
15	**0.4580**
20	**0.6106**
30	**0.9160**
50	**1.5267**
75	**2.2900**
100	**3.0534**

Figure S5. IC50 values for the GNP-MDS-PLU (Green line) and MDS free (Blue line) treated MV-4-11 Luc2 cells. The X axis represents the LogX of each concentration (ug/mL) that was evaluated and the Y axis represents represents the percentages of viability. Table illustrating the conversion between the treatment dosage expressed in volume units and the equivalent quantity of the MDS drug (µg) from the nanoparticle formulation.

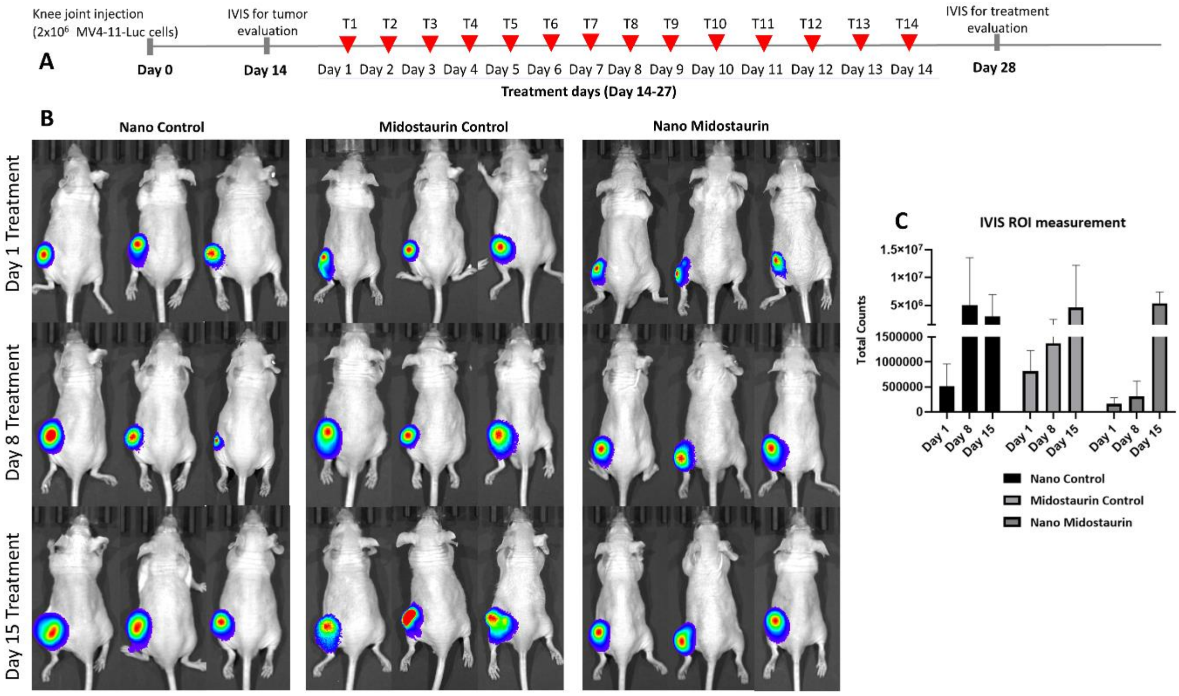

Figure S6. In vivo evaluation of systemic efficiency of various gold nanocarriers formulations–14 days of continued acute myeloid leukemia treatment. A) Scheme of tumour development, treatment administration and imagistic evaluation schedule. B) In vivo bioluminescent evaluation of mice grafted with MV4-11-Luc cells in the knee joint divided into three experimental groups: Nano Control, Midostaurin Control and Nano Midostaurin. IVIS imagining was done before treatment administration (Day 1 Treatment), after the first seven doses of treatment (Day 8 Treatment) and after the last dose of treatment (Day 15 Treatment). C) Graphical representation of the bioluminescent signal intensity within the region of interest (ROI) of the tumour graft before treatment administration (Day 1), after the first seven doses of treatment (Day 8) and after the last dose of treatment (Day 15) of the three experimental groups: Nano Control, Midostaurin Control and Nano Midostaurin.

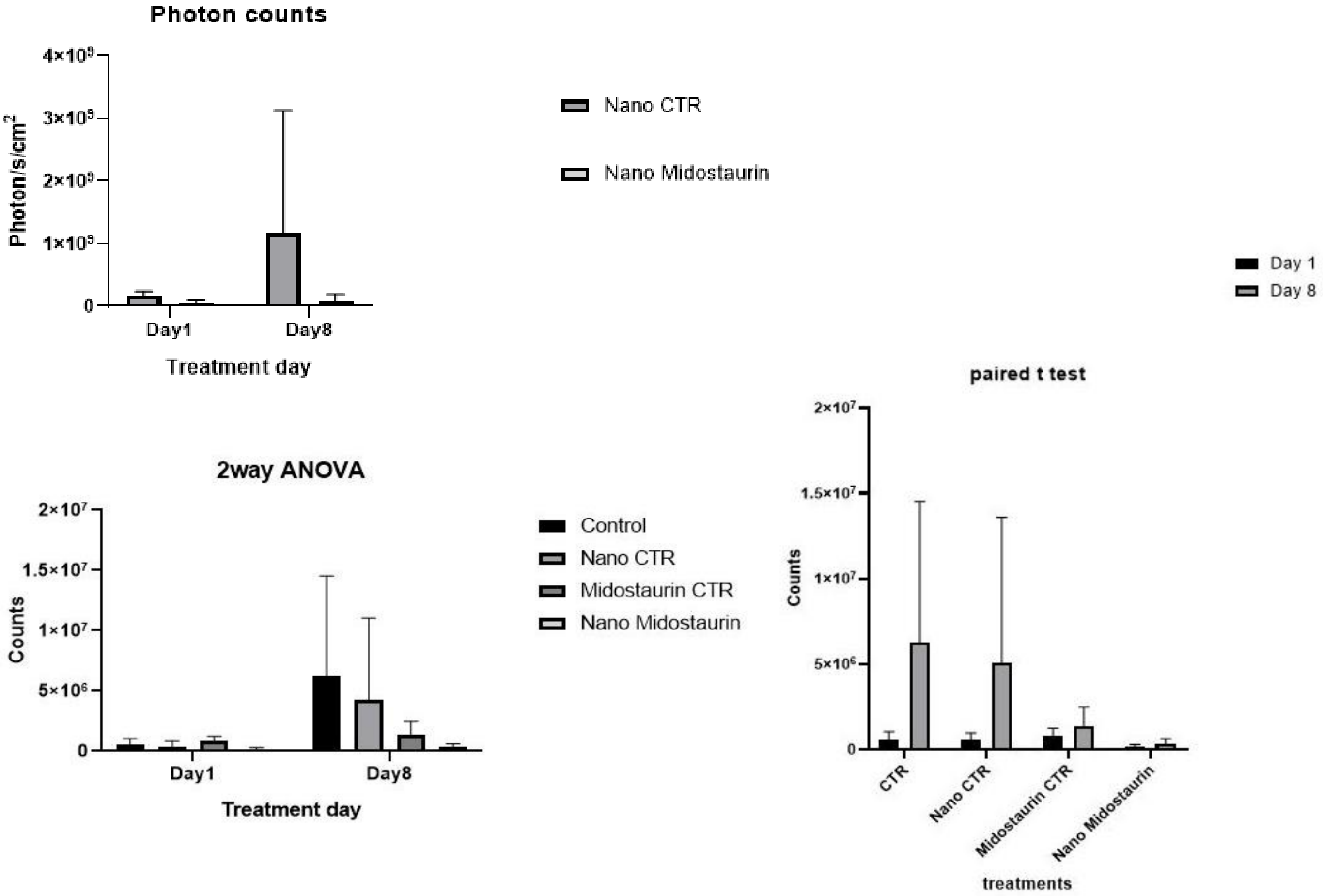

Figure S7. The p-values from the statistical tests (ANOVA, t-test) indicating trends toward improved efficacy of the nanoparticle-mediated Midostaurin delivery (no definite statistical significance is observed).
